# Exfoliation of large-area transition metal chalcogenide single layers

**DOI:** 10.1038/srep14714

**Published:** 2015-10-07

**Authors:** Gábor Zsolt Magda, János Pető, Gergely Dobrik, Chanyong Hwang, László P. Biró, Levente Tapasztó

**Affiliations:** 1 Centre for Energy Research, Institute of Technical Physics and Materials Science, 2D Nanoelectronics “Lendület” Research Group, Budapest, Hungary; 2 Korea Research Institute of Standards and Science, Center for Nanometrology, Daejeon, South Korea; 3 Centre for Energy Research, Institute of Technical Physics and Materials Science, Nanotechnology Department, Budapest, Hungary

## Abstract

Isolating large-areas of atomically thin transition metal chalcogenide crystals is an important but challenging task. The mechanical exfoliation technique can provide single layers of the highest structural quality, enabling to study their pristine properties and ultimate device performance. However, a major drawback of the technique is the low yield and small (typically < 10 μm) lateral size of the produced single layers. Here, we report a novel mechanical exfoliation technique, based on chemically enhanced adhesion, yielding MoS_2_ single layers with typical lateral sizes of several hundreds of microns. The idea is to exploit the chemical affinity of the sulfur atoms that can bind more strongly to a gold surface than the neighboring layers of the bulk MoS_2_ crystal. Moreover, we found that our exfoliation process is not specific to MoS_2_, but can be generally applied for various layered chalcogenides including selenites and tellurides, providing an easy access to large-area 2D crystals for the whole class of layered transition metal chalcogenides.

Layered transition metal chalcogenides (TMCs) display strong intra-layer metal-chalcogenide bonds, and a weak inter-layer bonding between neighboring planes of chalcogenide atoms^1^. The recent interest in studying their two-dimensional (2D) form (consisting of triple to quintuple atomic sheets) is driven by the fact that the properties of the atomically thin crystals can drastically differ from their well-characterized bulk counterparts. An eloquent example is the transition in the MoS_2_ band structure from indirect to direct bandgap as the number of layers is reduced from bulk to a single layer^2^ opening the way towards optoelectronic applications^3^. Furthermore, the family of transition metal chalcogenides is large, covering a broad range of properties from semiconductors (MoS_2_, WSe_2_) to semimetals (TiS_2_, TiSe_2_), from topological insulators (Bi_2_Te_3_, Bi_2_Se_3_) to correlated materials (NbS_2_, NbSe_2_). Such large variety of properties holds a huge potential for both fundamental studies and applications, even graphene cannot compete with in spite of the unique versatility of its properties.

The easy access to large TMC single layers is of key importance for exploring their properties, in a similar manner as the facile isolation of large and high-quality graphene flakes enabled the outstanding pace of the graphene research. As several bulk TMC crystals are layered materials, similar to graphite, individual TMC layers can be isolated by mechanical exfoliation^4^. However, by using the most widely-spread micromechanical cleavage (“scotch-tape”) technique, the lateral size of the exfoliated TMC single layers is typically of the order of micrometers^2^,^3^,^4^,^5^, which is about an order of magnitude smaller than the lateral size of the graphene flakes that can be routinely obtained by the same technique. The reason for this probably originates from the unique mechanical strength^6^ and ultra-strong adhesion^7^ of graphene to SiO_2_ that cannot be matched by the otherwise still excellent mechanical properties of TMCs^8^.

Alternative methods for producing single layers of various TMC materials are the chemical^9^,^10^ and liquid-phase exfoliation^11^ of single layers from their bulk crystals, as well as the Chemical Vapor Deposition (CVD) growth^12^,^13^ of TMC layers. The CVD technique in principle enables the growth of macroscopic areas of TMC films; however it is still difficult to grow single layers continuous on a macroscopic scale. Furthermore, the CVD growth of various TMC materials requires the development of a dedicated experimental setup and optimization for the growth of each TMC material separately. Chemically exfoliated TMC layers usually undergo irreversible chemical modification of their structure and properties and their lateral size is generally below 1 micron^9^. Recently, the electrochemical exfoliation of MoS_2_ flakes with up to 50 microns lateral size has been demonstrated; however even these layers undergo a partial oxidation^10^. Sonication assisted liquid phase exfoliation in various solvents yields large quantities of such exfoliated TMC layers, but most of the flakes are multilayers and their lateral size is typically of order of only a few hundreds of nanometers.

Compared to other methods, mechanical exfoliation provides 2D TMC sheets of high structural quality enabling the fundamental study of their pristine properties, and ultimate device performance, similar to graphene, where most of the fundamental discoveries have been achieved on exfoliated samples, owing to their superior structural and electronic quality^14^,^15^. The major limitation of the micromechanical exfoliation of various TMC materials is the small yield of single layers and their relatively small lateral size, typically of a few microns, rendering the subsequent investigations and device fabrication more difficult.

## Results

We have developed a novel mechanical exfoliation technique that overcomes the limitations of the scotch-tape technique enabling the exfoliation of TMC single layers with lateral size in the range of hundreds of microns. During the mechanical exfoliation process, the isolation of single layers is possible because the adhesion of the bottom layer to the substrate becomes stronger than the adhesion to its own bulk crystal. Graphene displays an ultra-strong adhesion to SiO_2_^7^, which can play an important role during the exfoliation process, facilitating the exfoliation of large (typically tens of microns) single layers. To improve the exfoliation process of MoS_2_ single layers we propose to improve their adhesion to the substrate. To achieve this, instead of the usually employed SiO_2_/Si, we used gold substrates exploiting the chemical affinity of the sulfur to gold^16^ to achieve a stronger adhesion. The gold substrates with large, atomically flat and clean Au (111) surfaces have been prepared by epitaxial growth of about 100 nm thick gold films on mica that were freshly cleaved from the mica surface before the experiments^17^. Next, thick multilayer flakes had been peeled off from a bulk MoS_2_ crystal using a thermal release tape. These flakes had been placed on the top of the freshly cleaved gold substrates. By heating up the sample to 90 °C the thick MoS_2_ flakes have been released from the tape onto the gold substrate. We used a short ultrasonic treatment (70 W, 1–10 s) in acetone to remove the thick MoS_2_ flakes from the gold surface. We found that after a few seconds of sonication several thick flakes have been detached; however, underneath them, the last (bottom) MoS_2_ layer remained attached to the gold substrate.

**Figure 1:**
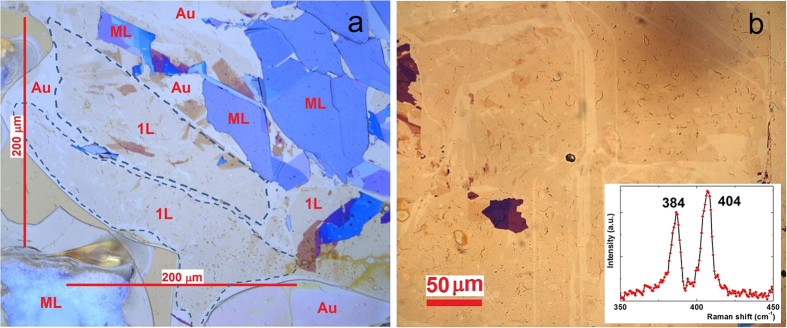
Mechanical exfoliation through chemically enhanced adhesion of large area MoS_2_ single layers. Optical microscopy images (**a**,**b**) of MoS_2_ single layer areas (1L, outlined by dotted lines) with several hundreds of microns lateral size exfoliated on gold (Au 111) substrate. The flakes of blue color are thick MoS_2_ multilayers (ML). The large areas of the faintest optical contrast have been confirmed to be single layers by Raman spectroscopy (inset).

**Figure 2:**
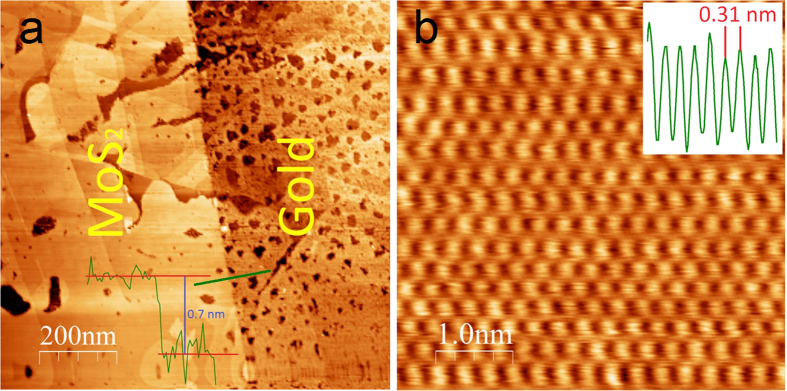
Atomic structure of MoS_2_ single layers. (**a**) STM image (500 mV, 1 nA) of a MoS_2_ single layer exfoliated onto a gold substrate. The relative height of the MoS_2_ layer was found to be 0.7 +/− 0.1 nm evidencing its single layer nature. The line cut displayed was taken along the direction marked by the green line across the edge. The darker areas of several tens of nanometers are atomic scale pits in the gold surface. (**b**) Atomic resolution STM image (5 mV, 2 nA) of a MoS_2_ single layer revealing a hexagonal lattice with 0.31 +/− 0.01 nm (see inset) periodicity corresponding to the crystal lattice of the top sulfur atoms.

In optical microscopy images the MoS_2_ single layers can be identified as the areas of the faintest color contrast as shown in Fig. 1. The optical images revealed several hundreds of microns large areas covered by thin MoS_2_ layers. To confirm that these areas of faintest optical contrast are indeed single layers of MoS_2_ we have performed confocal Raman spectroscopy measurements (λ = 532 nm, 1 mW) as shown in the inset of Fig. 1b. We have observed the characteristic MoS_2_ peaks^18^ (E_2g_, A_1g_) around 384 and 404 cm^−1^, wavenumbers respectively. This indicates that the several hundreds of microns large areas observed by optical microscopy can be identified as MoS_2_ single layers^4^,^18^,^19^. The exfoliated large flakes on top of the conductive Au substrate enabled us to perform Scanning Tunneling Microscopy (STM) measurements on mechanically exfoliated single layer MoS_2_ flakes. The height of the investigated layers relative to the Au substrate was found to be about 0.7 +/− 0.1 nm from the STM measurements (Fig. 2a), confirming the single layer thickness of the exfoliated flakes. Atomic resolution images could be routinely achieved even under ambient conditions (Fig. 2b). A hexagonal atomic lattice has been revealed with 3.1 +/− 0.1 Å periodicity, corresponding to the lattice constant of the top layer of sulfur atoms^20^.

**Figure 3:**
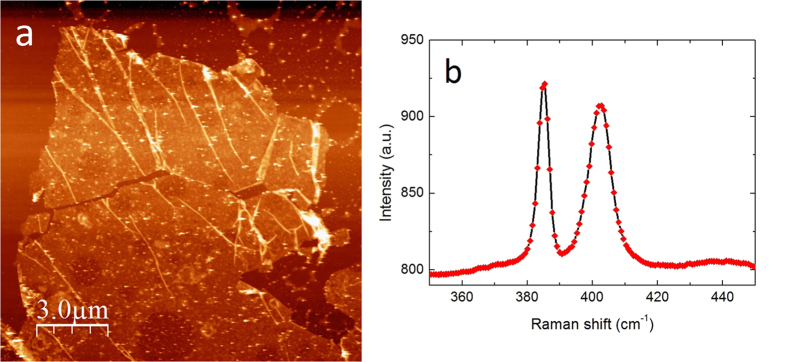
MoS_2_ single layers on insulating substrate. (**a**) Atomic Force Microscopy image of a MoS_2_ layer exfoliated to gold and transferred to a SiO_2_/Si substrate. (**b**) Raman spectra (λ = 532 nm, 1 mW) of the transferred single layers evidence that MoS_2_ layers can be reversibly detached from the Au substrate without modifying their structure.

While for STM measurements the gold substrate is ideal, several experimental techniques including electrical transport measurements and device applications require insulating substrates. Furthermore, it is important to get an idea about the nature of the bonding between the MoS_2_ single layers and the gold substrate, and most importantly whether it is fully reversible or not. Raman spectroscopy and atomic resolution STM measurements revealed no significant deviation from the intrinsic structure of MoS_2_. However, to fully clarify this issue we have transferred the exfoliated MoS_2_ single layers from gold to SiO_2_/Si substrate for further investigations. To realize this, a thermal release tape has been placed on top of the exfoliated MoS_2_/Au sample. Then, using potassium iodide solution we etched away the gold substrate^21^. The MoS_2_ flakes supported by the thermal release tape can then be transferred to any desired substrate. Upon heating up the sample the flakes are released onto the surface of the new substrate; in our case SiO_2_/Si. An atomic force microscopy image of a transferred MoS_2_ flake on top of SiO_2_/Si is shown in Fig. 3a. The Raman spectra of the MoS_2_ flakes transferred from gold to SiO_2_/Si (Fig. 3b) perfectly match the characteristic spectra of MoS_2_ single layers directly exfoliated to SiO_2_/Si substrates^18^,^19^, indicating that MoS_2_ layers can be reversibly detached from the gold surface after exfoliation, without significant structural modification.

**Figure 4:**
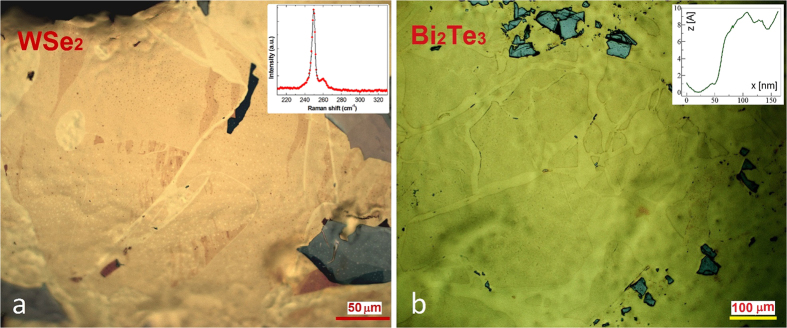
Exfoliation of various layered chalcogenide single layers. Optical microscopy images of WSe_2_ (**a**) and Bi_2_Te_3_ (**b**) single layers. The insets display the Raman spectrum of WSe_2_ (**a**) and AFM line cut over a Bi_2_Te_3_ step edge (**b**) confirming the single layer nature of the exfoliated large area flakes.

We have also investigated whether the exfoliation process is specific to MoS_2_ or can be applied more generally to various layered materials. We found that our exfoliation method yielding large-area MoS_2_ flakes is not specific to molybdenum disulfide or even sulfides, but works equally well for various layered chalcogenides, including selenides and tellurides. To illustrate this in Fig. 4 we present optical microscopy images of WSe_2_, and Bi_2_Te_3_ single layers with several hundreds of microns lateral scale. However, when we attempted to apply the technique for the exfoliation of graphene, we found that after a short sonication all thick graphite flakes have been removed from the gold substrate leaving behind no graphene layers at all. This further supports that the mechanism behind our exfoliation process is indeed the chemically enhanced adhesion of various layered TMC materials. Furthermore, in contrast to the standard scotch-tape method, the exfoliation technique reported here is in principle able to achieve high coverage rates of macroscopic substrates with various TMC single layers.

In conclusion, we have demonstrated a simple and general method to isolate large-area single layers of various layered transition metal chalcogenides exploiting their chemically enhanced adhesion to gold substrates, opening the way towards the systematic exploration of the novel properties of two dimensional crystals form the whole family of layered chalcogenides.

## Methods

Optical imaging has been performed by a Zeiss Axio Imager microscope. Scanning Tunneling Microcopy measurements have been performed on a Nanoscope E instrument under ambient conditions. Atomic resolution images could be routinely achieved with the typical settings of U_b_ = 5–100 mV bias voltage and I_t_ = 1–3 nA tunneling current. Atomic Force Microscopy images have been acquired by a Bruker Multimode 8 AFM, in tapping mode. The contaminations revealed by AFM on the MoS_2_ flakes transferred to SiO_2_/Si substrate are most probably gold nanoparticle residues from the wet etching process of the gold substrate. Raman spectra have been acquired by a Witec 300RSA confocal Raman microscope using 532 nm laser wavelength, and typical powers of 0.2–2 mW. Raman spectroscopy was also used to confirm the single layer nature of WSe_2_ flakes, while atomically thin Bi_2_Te_3_ layers were found to be very unstable even for low (0.2 mW) laser powers. We found it important to use freshly cleaved stripped gold substrates; however, their source and flame annealing was not crucial for achieving good results. Commercially available thermal release tape has been used for all transfer experiments.

## Additional Information

**How to cite this article**: Magda, G. Z. *et al.* Exfoliation of large-area transition metal chalcogenide single layers. *Sci. Rep.* **5**, 14714; doi: 10.1038/srep14714 (2015).
